# Enhanced Hole Injection Characteristics of a Top Emission Organic Light-Emitting Diode with Pure Aluminum Anode

**DOI:** 10.3390/nano11112869

**Published:** 2021-10-27

**Authors:** Chan Young Park, Byoungdeog Choi

**Affiliations:** Department of Electrical and Computer Engineering, Sungkyunkwan University, 2066 Seobu-Ro, Jangan-Gu, Suwon 16419, Korea; parkcy@skku.edu

**Keywords:** OLED, top emitting, hole injection, Al anode, ITO anode, HATCN, efficiency, native oxide, microdisplay, Si substrate

## Abstract

A top emitting organic light-emitting diode (OLED) device with pure aluminum (Al) anode for high-resolution microdisplays was proposed and fabricated. The low work function of the Al anode, even with a native oxide formed on the Al anode surface, increases the energy barrier of the interface between the anode and hole injection layer, and has poor hole-injection properties, which causes the low efficiency of the device. To enhance the hole-injection characteristics of the Al anode, we applied hexaazatriphenylene hexacarbonitrile (HATCN) as the hole-injection layer material. The proposed OLED device with a pure Al anode and native oxide on the anode surface improved efficiency by up to 35 cd/A at 1000 nit, which is 78% of the level of normal OLEDs with indium tin oxide (ITO) anode.

## 1. Introduction

Organic light-emitting diodes (OLEDs) are currently applied to display panels for smart phones, tablets, and TVs. The characteristics of OLEDs, such as their being self-emitting, and having a thin form factor, vivid color, and fast response time, attract attention for use in display panels and various other applications. However, OLEDs have a limited resolution caused by the fabrication method of pixel-patterning using a fine metal mask (FMM), which is a shadow mask with tiny holes for patterning pixels from evaporated organic materials. The resolution of an OLED display panel using FMM is approximately 800 ppi at present [1]. For a microdisplay, which has high resolution of over 2000 ppi, applied to Virtual Reality (VR) devices and Augmented Reality (AR) devices, the FMM fabrication method is unavailable because the hole size of FMM is smaller than 10 um. Another approach was used to fabricate OLED microdisplays, involving a separated backplane and OLED panel [2,3]. The backplane driving each pixel is made of a single crystal silicon wafer substrate with a complementary metal-oxide-semiconductor (CMOS) process at the semiconductor foundry. OLEDs are fabricated on the wafer backplane using the evaporation process. Instead of separated RGB pixels, white OLED-stacked RGB organic layers, and RGB color filters corresponding to each pixel, are used for color patterning OLED microdisplays [3].

Since the wafer backplane is not transparent, as opposed to the glass substrate normally used in the display device process, a top emitting OLED structure has to be applied to OLED microdisplays [4]. In top emitting OLEDs, the anode materials should be highly reflective in the visible range to achieve a high luminance efficiency, and should have a high work function for the efficient injection of holes. Many previous studies have been carried out on the anode for top emitting OLEDs. Silver (Ag) has a high reflectivity of 94% at wavelengths of 520 nm and a low electrical resistivity of 1.47 μΩ cm at a temperature of 298 K [5]. An Ag/indium tin oxide (ITO) composite bi-layer structure combined the advantages of both the high reflectivity of Ag and the high work function of ITO [6,7]. Other materials have been studied for use as the anode in top emitting OLEDs such as aluminum (Al)/ITO [8], Ag/Al [9], gold (Au) [10], molybdenum (Mo) [11], platinum (Pt) [12], and vanadium (V) [13]. However, despite their good electrical and optical characteristics, the materials mentioned above are not supported by the current general CMOS foundry. Al, titanium (Ti), and Tungsten (W) are available as the anode for top emitting OLEDs at the general CMOS foundry. Some approaches have been studied that use available materials as the anode of top emitting OLEDs, supported by a general CMOS foundry, such as TiN, Al/TiN bi-layer [14,15,16]. However, top emitting OLEDs with Ti and TiN anodes have limited efficiency due to the low reflectivity of the anode. Other approaches have been studied, applying Al as the anode of top emitting OLEDs using oxide buffer materials [17] or inverted OLED structures [18,19]. Al has a high reflectance that leads to highly efficient top emitting OLEDs. However, Al has a low work function of 4.2 eV compared to ITO, with a work function of 4.7 eV, which is normally used as the OLEDs anode. The energy barrier between the low-work-function anode and hole-injection layer (HIL) in OLEDs is higher, so poor hole-injection characteristics decrease the efficiency of OLED devices. In addition, the native oxide (Al_2_O_3_) formed on the Al anode surface, decreases efficiency.

In this study, we proposed a top emitting OLED device structure with an Al anode. To enhance the hole-injection characteristics of the Al anode, we applied hexaazatriphenylene hexacarbonitrile (HATCN) as the HIL material. The fabricated top emitting OLED devices, with enhanced hole-injection characteristics, improved the efficiency of normal OLEDs with an ITO anode by 78%.

## 2. Materials and Methods

**Figure 1:**
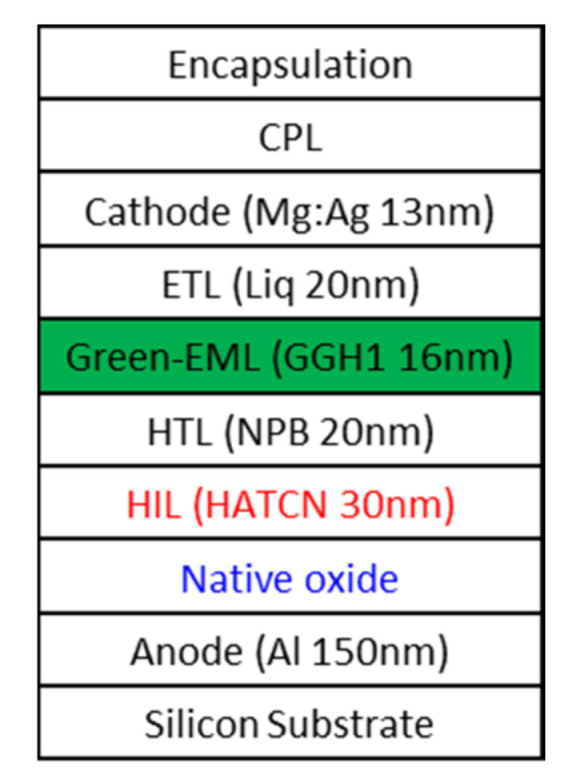
Schematic diagram of proposed structure of green top emitting OLED device.

Figure 1 presents the schematic structure of the device. In the OLED microdisplay, the backplane fabrication process and OLED fabrication process are separated. Therefore, the backplane was fabricated at the general CMOS foundry until the Al anode layer formed. The OLED was fabricated at a different site, with the Al anode formed backplane, from HIL to capping layer (CPL) using the thermal evaporation method. During the transport from the foundry to OLED evaporation site, the Al anode had to be exposed to air, causing native oxide on the surface of Al anode. We used 8-inch (200 mm) silicon wafers as substrate at DB HiTek (Bucheon, Korea). Al of 150 nm thickness was deposited on the substrate using the sputter method for a reflective anode. We stored the Al-deposited substrate in a clean room for one day to purposefully form native oxide, assuming an extreme case. We then deposited a HIL, hole-transport layer (HTL), green emission layer (EML), electron-transport layer (ETL), cathode, and CPL in sequence using the thermal vacuum evaporation method. We used hexaazatriphenylene hexacarbonitrile (HATCN) of 30 nm thickness as the HIL, N′-bis(phenyl)benzidine (NPB) of 20 nm thickness as the HTL. GGH1 (Gracel, Korea) of 16 nm thickness was used as green EML and hydroxyquinolatolithium (Liq) of 20 nm of thickness was used as ETL. Mg:Ag of 13 nm thickness was used as the semi-transparent cathode. Most materials used in the devices were provided by Sigma-Aldrich (St. Louis, MO, USA) as commercial-grade powder sources, except GGH1, from Cracel (Seoul, Korea). The total thickness of the OLED device between the reflective anode and semi-transparent cathode was decided as 86 nm as in (1) to improve the efficiency using a micro-cavity effect [20]. The thickness of the layers of the devices was determined in situ by the crystal monitor in the vacuum chamber. We also fabricated an ITO anode OLED device for reference to compare the characteristics of OLEDs between Al anode and ITO anode

(1) $$I_{\mathrm{cav}}\left(\lambda \right)=\frac{\left(1-R_{2}\right)\left[1+R_{1}+2{\left(R_{1}\right)}^{0.5}\cos\left(\frac{4\pi d}{\lambda }\right)\right]}{1+R_{1}R_{2}-2{\left(R_{1}R_{2}\right)}^{0.5}\cos\left(\frac{4\pi L}{\lambda }\right)}\times I_{\mathrm{nc}}\left(\lambda \right)$$

where *I*_cav_ is the spectrum intensity, *R*_1_ and *R*_2_ are the reflectivities of the anode and cathode mirrors, respectively, d is the effective distance of the emitting layer from the anode, L is the total optical thickness of the cavity, and *I*_nc_ is the free space electroluminescence intensity at wavelength λ.

Measurements of OLED properties were performed by recording current–voltage characteristics as well as electro-luminescence (EL) spectra. The current–voltage was acquired using a Keithley 236 voltage source unit, while the EL intensity, luminance and spectral characteristics of the devices were measured with a calibrated silicon photodiode (Hamamatsu Photonics, Hamamatsu, Japan, S5227-1010BQ), a photomultiplier tube, and a spectroradiometer (Minolta, Osaka, Japan, CS-1000).

## 3. Results and Discussion

The characteristics of the OLED device with an Al anode are presented in Table 1. The current efficiency of the OLED device is 35 cd/A at 1000 nit, which is 78% of the conventional OLED device with an ITO anode. Usually, the efficiency of OLEDs with an Al anode is below 50% of OLEDs with an ITO anode [21]. The proposed OLED device with an Al anode has high efficiency, even though native oxide was formed on the Al anode. This means that hole injection characteristics of the proposed OLED device are enhanced by Al anode and HATCN HIL, even though an energy barrier exists between the Al anode and HATCN HIL.

**Figure 2:**
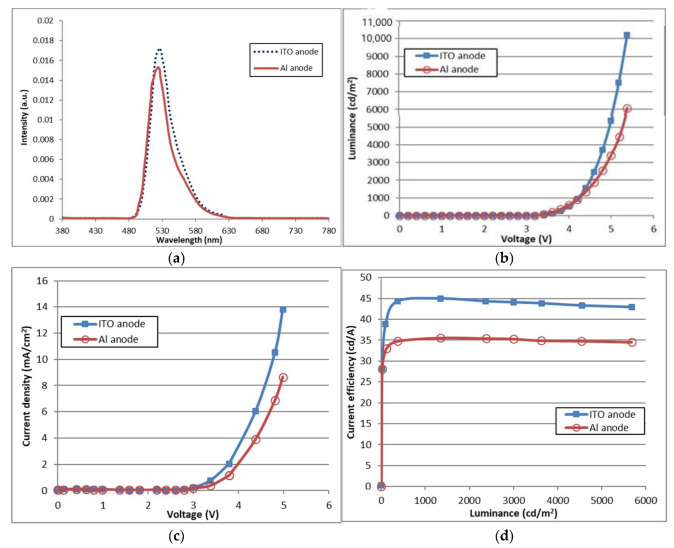
Characteristics of OLED devices with Al anode and HATCN HIL: (**a**) Electroluminescence spectrum of the OLED devices; (**b**) Characteristics of luminance for different voltages; (**c**) Characteristics of current density for different voltages; and (**d**) Characteristics of current efficiency for difference luminance.

Figure 2 shows the optical and electrical characteristics of the OLED devices with an Al anode. In the spectrum of OLED devices, the main peak in the spectrum, at 525 nm, was not changed by the anode materials. The electroluminescence spectrum of the OLED devices is determined by the thickness of the OLED device. Therefore, the spectrum is not related to the hole-injection characteristics of the OLED device. The driving voltage of the OLED device with an Al anode was 4.2 V, an increase of 0.4 V compared to the OLED device with an ITO anode at 10 mA/cm^2^. The increase in voltage is caused by native oxide in the Al anode, which increases the resistance of the interface between the anode and HIL. The power consumption of the OLED device with an Al anode can be increased slightly, but this does not matter when operating an OLED microdisplay panel with an Al anode. The current efficiency of the OLED device with an Al anode was 35 cd/A at 1000 nit, which is 78% of the current efficiency of the OLED device with an ITO anode at 44 cd/A.

**Figure 3:**
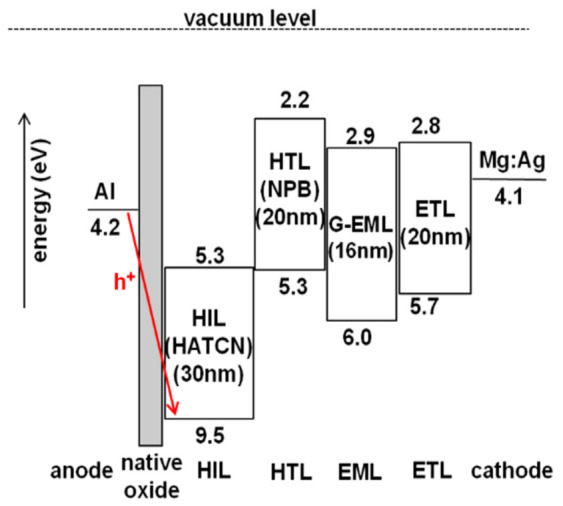
Energy band diagram of the proposed OLED device with Al anode and HATCN HIL.

Figure 3 shows the energy band diagram of each of the layers comprising the proposed OLED device. As shown in Figure 3, there is a high energy barrier between the Al anode and HIL, so it is hard to inject holes from the Al anode, with a low work function of 4.2 eV, to the HATCN HIL at 9.5 eV, which is the highest occupied molecular orbital (HOMO). However, the proposed OLED device shows high efficiency with the low-work-function Al anode, even with native oxide on the surface of the Al anode. This can be explained by the charge generation at the HATCN/NPB interface and the high electron-withdrawing properties of HATCN to the anode [22,23,24]. HATCN has good electron-transport characteristics. At the interface of HATCN and NPB, the energy level difference between the lowest occupied molecular orbital (LUMO) of HATCN and HOMO of NPB is small enough for electrons in the HOMO of NPB to move to the LUMO of HATCN. Moreover, while the electron-holed pairs are generated at the interface of HATCN and NPB, HATCN constantly transports electrons to the Al anode. Since the electrons are effectively transported from HATCN to the Al anode, the hole-injection property is enhanced. Therefore, HATCN improved hole-injection characteristics from the Al anode to HIL in the proposed OLED structure.

## 4. Conclusions

We investigated the characteristics of OLED devices with a reflective Al anode and fabricated a high-efficiency, green top emitting OLED device with an Al anode by improving the hole-injection characteristics. We obtained an efficiency of 35 cd/A at 1000 nit from the OLED with an Al anode, which is 78% of a normal OLED with an ITO anode. Using HATCN as the HIL improved the hole-injection characteristics of the low-work-function Al anode, even though native oxide had formed on the Al anode surface, which increased the energy barrier of the interface between the Al anode and HIL. This result indicates that a high-efficiency OLED microdisplay using an Al anode for high-resolution patterning can be fabricated using the enhanced hole-injection structure of an OLED device. Thus, we believe that this study can provide a simple, practical, and low-cost method for improving the performance of OLED microdisplay products in the current foundry infrastructure.

## Figures and Tables

**Table 1:** Measured characteristics of green top emitting OLED devices with Al anode and ITO anode.

Parameters	Device with Al Anode	Device with ITO Anode	Notes
Current efficiency (cd/A)	35	45	@1000 nit
Driving voltage (V)	4.2	3.8	@1000 nit
Peak wavelength (nm)	525	525	
CIE color coordination	(0.22, 0.71)	(0.23, 0.71)	
